# Assessing Quality of Life and Medical Care in Chronic Angina: An Internet Survey

**DOI:** 10.2196/ijmr.4971

**Published:** 2016-04-28

**Authors:** Karen P Alexander, Olena Stadnyuk, Suzanne V Arnold, Daniel B Mark, E. Magnus Ohman, Kevin J Anstrom

**Affiliations:** ^1^ Duke University Medical Center Department of Cardiology Durham, NC United States; ^2^ Duke Clinical Research Institute Durham, NC United States; ^3^ Gilead Therapeutics Foster City, CA United States; ^4^ Mid America Heart Institute St. Louis, MO United States; ^5^ Duke Hospital Duke Program for Advanced Coronary Disease Durham, NC United States

**Keywords:** Angina, Surveys and Questionnaires, Internet, Quality of Life

## Abstract

**Background:**

Angina is a clinical syndrome whose recognition relies heavily on self-report, so its identification can be challenging. Most data come from cohorts identified by physicians and nurses at the point of care; however, current widespread access to the Internet makes identification of community cohorts feasible and offers a complementary picture of angina.

**Objective:**

To describe a population self-identified as experiencing chronic angina by use of an Internet survey.

**Methods:**

Using email and an Internet portal, we invited individuals with a diagnosis of angina and recent symptoms to complete an Internet survey on treatment and quality of life (QOL). In total, 1147 surveys were received. The main analysis was further limited to those reporting a definite coronary heart disease (CHD) history (N=646, 56% of overall).

**Results:**

Overall, about 15% reported daily angina and 40% weekly angina. Those with more frequent angina were younger, more often depressed, and reported a shorter time since diagnosis. They also had substantially worse treatment satisfaction, physical function, and overall QOL. Fewer than 40% were on ≥ 2 anti-anginals, even with daily angina. The subjects without a history of definite CHD had unexpectedly low use of antianginal and evidence-based medicines, suggesting either a lack of specificity in the use of self-reported angina to identify patients with CHD or lack of access to care.

**Conclusions:**

Use of inexpensive electronic tools can identify community-based angina cohorts for clinical research. Limitation to subjects with a definite history of CHD lends diagnostic face validity to the approach; however, other symptomatic individuals are also identified.

## Introduction

Angina is a clinical syndrome whose recognition relies heavily on self-report. The subjective nature of angina challenges its efficient recognition and proper identification. The prevalence of angina has been estimated to be ~3.2% among US adults >20 years of age and 8-10% among those aged over 60 years old [1]. In addition, among trials and registry populations of patients with coronary artery disease, 20-30% continue to report angina symptoms despite contemporary medical care [2-8]. Since angina is associated with risk for morbidity, poor quality of life (QOL), and increased health care costs, evidence suggesting poor control of symptoms is concerning [9-11]. The diagnosis still rests upon patient report, with no fully satisfactory method of objective confirmation yet accepted. Despite these difficulties, chest discomfort symptoms remain a huge source of patient distress, and incomplete control may be an important signal of inadequate quality of care. The use of electronic tools on the Internet, including email and patient surveys, provides potentially useful new methods to survey large cohorts and identify unmet needs deserving of more intensive study.

We postulated that we could identify a cohort of individuals with a diagnosis of angina and recent symptoms via Internet and email links that could provide some insights into the levels and effectiveness of care from the patient’s perspective. We were specifically interested in the group with greater symptom burden, to understand the impact of their angina on treatment and QOL.

## Methods

### Participants

Participants were identified through email and the Internet. Direct-to-patient emails with survey links were sent to two advocacy LISTSERVs maintained by professional organizations: Mended Hearts Inc., a cardiovascular patient membership organization, and the American College of Cardiology (ACC) Vendor “Opt-in” cardiovascular patient LISTSERV. In addition, a Web-based survey was available through an Internet link following search engine inquiries for “angina” or “angina treatment” and on the webpage of the Society for Cardiovascular Angiography and Interventions. [12] Unique US IP addresses were required. In both the email and the Internet link, a brief description of the survey was provided, and respondents opted in by clicking on the survey URL.

The target respondents were individuals aged ≥50 years who had a diagnosis of angina at any time in the past and also experienced angina in the prior 6 months. The survey was distributed during an 8-week period from November 2012 to January 2013. Respondents were invited to complete the survey if they met the eligibility by indicating yes to each criterion in the screening survey (age ≥50 years, provider diagnosis of angina, and occurrence of angina in the past 6 months). A description of angina was provided: “Angina is a pain, discomfort, or pressure localized in the chest that is caused by an insufficient supply of blood (ischemia) to the heart muscle. It is also sometimes characterized by a feeling of choking, suffocation, or crushing heaviness. This condition is called angina pectoris.” Respondents were excluded if they were employed or had an immediate family member employed by a marketing/research organization, or if they had participated in a research study on angina in the last 6 months. As an incentive, survey participants were offered entry into a drawing to win one of five $100 gift cards.

### Survey

The survey was composed of 34 questions and took approximately 15 minutes to complete. The survey included demographic and clinical factors, contact with health care providers, medication use, and health-related QOL (HRQOL): overall health, angina burden, physical functioning, and social and emotional well-being. Angina and disease-specific HRQOL items were assessed using the Seattle Angina Questionnaire (SAQ). The SAQ is a 19-item self-administered questionnaire that measures 5 clinically important dimensions of coronary disease: physical limitation, angina stability, angina frequency, treatment satisfaction, and disease perception [13]. Domain scores range from 0 to 100, with lower scores indicating more angina and worse QOL. The specific measure of angina frequency used to stratify patients for analysis was based on the following question: “Over the past 4 weeks, on average, how many times have you had to take nitroglycerin for chest pain, chest tightness, or angina?” Patients were grouped according to the following responses: daily, ≥4 times daily, and 1-3 times daily; weekly, >3 times weekly but not daily, and 1-2 times per week; none/monthly, < once a week or no angina in last 4 weeks.

Global health status was assessed using the Global Health Question from the RAND Medical Outcomes Study (MOS) questionnaire, the EuroQol-5D questionnaire (EQ-5D), and the Work/Regular Physical Activity questionnaire. The 5-item MOS questionnaire is a self-assessment of a patient’s overall general health, with responses ranging from excellent to poor [14]. The EQ-5D is a 5-item tool that assesses a patient’s perception of his or her mobility, self-care, usual activities, pain/discomfort, and anxiety/depression [15]. EQ-5D responses can be converted to a standard scale using an algorithm developed for the US population [16], which range from 0 to 1, with 0 representing the worst imaginable health state and 1 representing perfect health [15]. The Work/Regular Physical Activity questionnaire is a 1-item questionnaire that assesses how active a subject is at work (including volunteer work and housework) [17]. The possible ordinal responses to the question are 1 = Mainly sedentary, 2 = Predominantly walking on one level, 3 = No heavy lifting, 4 = Mainly walking, including climbing stairs, walking uphill, or lifting heavy objects, 5 = Heavy physical labor, and 6 = Do not work. The response “Do not work” is not applicable to the physical activity at work endpoint, and therefore, individuals with this response were excluded from analysis of this domain of health.

### Analysis

Respondents confirmed that they had received a provider diagnosis of angina. However, the main analysis was further limited to respondents who also reported a confirmatory CHD history—who reported taking antiplatelet agents and had a prior percutaneous coronary intervention (PCI), prior coronary artery bypass grafting (CABG), or coronary heart disease diagnosis. Demographics, clinical history, comorbidities, general clinical care, and HRQOL were compared according to SAQ angina frequency groups. Supplementary analyses for the non-CHD confirmed cohort are shown in Multimedia Appendix 1, Multimedia Appendix 2, and Multimedia Appendix 3. The purpose of the analysis was descriptive and no specific hypotheses were prespecified. Analyses were performed using SAS Version 9.4.

All pages of the survey required complete answers prior to advancing; therefore, there were no missing items. As participants voluntarily submitted responses to this anonymous survey, the Duke institutional review board waived the need for consent. The study concept originated with the sponsor, Gilead Sciences. The sponsor contracted with members of the Outcomes Group at Duke Clinical Research Institute (DCRI) to design the survey, perform the analyses, and report the results. The DCRI retained full independence in matters of analysis, interpretation, and publication. The sponsor was given an opportunity to review and comment on the publication prior to submission, but final responsibility for content remained with DCRI authors. One sponsor representative who worked with DCRI on the study design was included as a coauthor.

## Results

**Figure 1:**
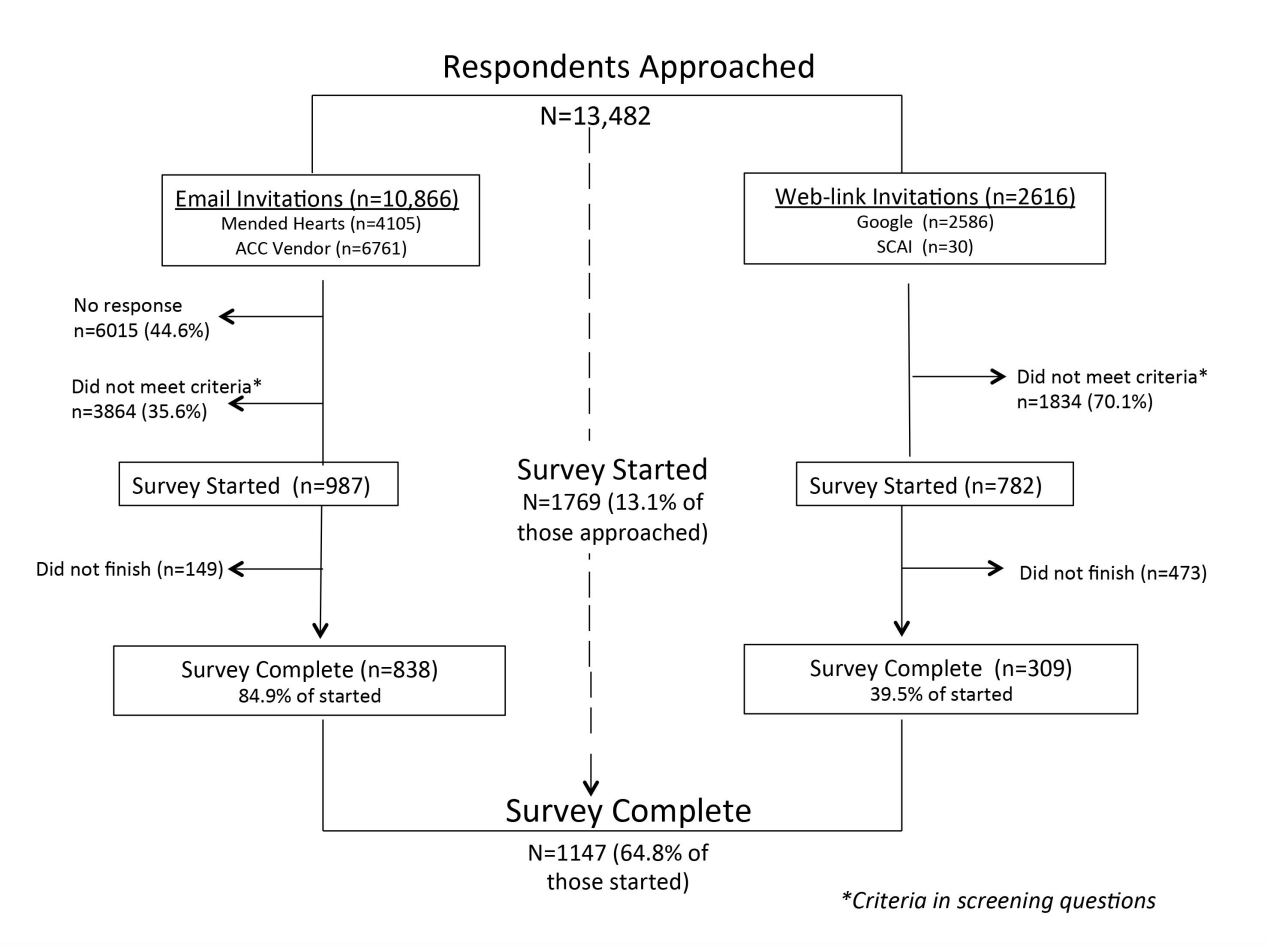
Flow diagram of respondents by invitation and completion.

A total of 13,482 individuals were approached to complete the survey (10,866 through email, 2616 through an Internet link), of which 44.6% (6015/13,482) did not respond (Figure 1). Screening questions excluded 76.3% (5698/7467) (respondents who initiated the screening phase. In the email survey invitation group (half of emails received no response), 35.6% did not meet screening criteria, and 84.9% of those who did meet criteria completed the survey. In the Internet link group who had clicked on the survey invitation, 70% (1834/2616) did not meet screening criteria, and 39.5% (309/782) of those who did meet criteria completed the survey. Of the 1769 respondents who passed screening criteria, an additional 622 did not complete the survey. This left 1147 complete survey respondents; 73% (838/1147) from email invitations and 27% (309/1147) from Internet invitations—a response rate that exceeded the anticipated target of 1000 patients in 8 weeks. The survey was completed by 64.8% (1147/1769) of those respondents who started it, and 9% (1147/13,482) of all those approached.

Of the 1147 respondents with completed surveys, 73% were from obtained from email and 27% from Internet contact (Table 1). Email respondents were more often male, with higher educational attainment, and longer time since first angina diagnosis compared to Internet respondents. Over half of Internet respondents reported a diagnosis of angina within the last year in contrast to 15.5% of email respondents. Also, more Internet respondents said their angina diagnosis date was unknown. Uncertainty about cardiovascular disease among Internet respondents was also suggested by fewer visits to a cardiologist, lower rates of having a cardiologist, and less prior revascularization. Therefore, the primary analysis was limited to those reporting use of a daily antiplatelet and a prior PCI, CABG, or CHD history (CHD group). The CHD group comprised over half of survey respondents (N=646, 56%). The CHD group more often came from email (n=544) than from Internet (n=102) invitations.

Demographics and treatment of the CHD group stratified by self-reported angina frequency are shown in Table 2. Patients with more angina (daily) were younger and less likely to have a college education. Out-of-pocket medication costs and insurance coverage were similar across groups. Patients with more angina had notably more depression compared to those with less frequent or no angina.

Patients with more angina (daily) had a shorter time since initial angina diagnosis (Table 3). Most reported care by a cardiologist, and the majority discussed angina at their last visit. Differences in medication use revealed more long-acting nitrates and more anti-anginals for respondents with daily angina.

Subjects with self-reported angina but without definite CHD (non-CHD group) had lower use of anti-anginal and prevention medicines, even with daily angina, and more anxiety and depression (Multimedia Appendix 1). Over half were female, 9.6% were uninsured, and they were more likely to not have a cardiologist. In addition, symptom burden as reflected in the SAQ responses was nearly the same, and treatment satisfaction was lower in this group.

All disease-specific health status measures confirmed that QOL was substantially impaired in those reporting more angina (Table 4). As compared to those with no angina or monthly angina, those with daily angina had more angina-related physical limitations, worse disease-specific QOL, and lower angina-related treatment satisfaction. In addition, those reporting more angina had more problems in all 5 EQ-5D domains—particularly anxiety/depression, pain, and limitations in usual activities. Work/Regular Physical Activity questionnaire scores correlated with angina frequency, as respondents with more angina were less likely to report engaging in moderate to strenuous exercise.

**Table 1:** Demographics and characteristics by mode of invitation.

Variable		Email n=838	Internet n=309	*P* Value
Age, mean years ± SD		64.8 ± 8.3	64.1 ± 9.3	.095
Male, n (%)		511 (61.0)	155 (50.2)	.001
White race, n (%)		793 (94.6)	278 (90.0)	.005
Education (≥HS), n (%)		831 (99.2)	287 (92.9)	<.001
Married, n (%)		600 (71.6)	211 (68.3)	.27
Insurance, n (%)	Medicare/Medicaid	496 (59.2)	150 (48.5)	.001
	Private/employer	537 (64.1)	176 (57.0)	.028
	No insurance	28 (3.3)	33 (10.7)	<.001
Angina diagnosis, mean years ± SD		9.7 ± 8.9	3.7 ± 6.4	<.001
	<1 year (%)	130 (15.5)	175 (56.6)	
	1-5 years (%)	236 (28.2)	78 (25.2)	
	6-10 years (%)	185 (22.1)	22 (7.1)	
	>10 years (%)	287 (34.3)	34 (11.0)	
	Unknown (%)	27 (3.2)	27 (8.7)	
Cardiologist last 6 months	Yes	608 (72.6)	142 (46.0)	<.001
	No	183 (21.8)	103 (33.3)	
	Don’t have one	47 (5.6)	64 (20.7)	
Antiplatelet daily^a^		710 (84.7)	203 (65.7)	<.001
CHD^b^		438 (52.3)	87 (28.2)	<.001
Prior revascularization		518 (61.8)	98 (31.7)	<.001
	Prior PCI^c^	437 (52.2)	85 (27.5)	<.001
	Prior CABG^d^	288 (34.4)	30 (9.7)	<.001
Primary CHD group		544 (64.9)	102 (33.1)	<.001

^a^ Antiplatelet is either aspirin or other antiplatelet agent

^b^ CHD: coronary heart disease

^c^ PCI: percutaneous coronary intervention

^d^ CABG: coronary artery bypass grafting

**Table 2:** Demographics and conditions: overall and by angina frequency.

Variable		Overall N=646	Daily n=90	Weekly n=238	None/Monthly n=318	*P* Value	
Age, mean years (SD)		65.9 (8.3)	63.3 (8.0)	66.0 (8.1)	66.5 (8.4)		.005
Male, %		68.7	68.9	64.7	71.7		.21
White race, %		94.4	93.3	94.5	94.7		.89
Education ≥HS, %		98.5	95.6	99.2	98.7		.052
Married, %		74.8	77.8	72.7	75.5		.59
Insurance status	Medicare/Medicaid, %	61.6	52.2	67.7	59.8		.024
	Private/employer, %	64.6	75.6	58.4	66.0		.011
	No insurance, %	2.0	3.3	1.7	1.9		.62
Out-of-pocket prescription costs, %	1 (almost negligible)	27.6	20.0	26.5	30.5		.36
	2	26.0	26.7	25.6	26.1		
	3	27.1	28.9	25.6	27.7		
	4	14.7	18.9	16.0	12.6		
	5 (can’t fill all meds)	4.6	5.6	6.3	3.4		
Conditions, %	CHD^a^	76.0	81.1	77.3	73.6		.28
	Hypertension	63.0	64.4	60.5	64.5		.60
	Prior revascularization	88.2	87.8	85.7	90.3		.26
	Prior PCI^b^	74.9	74.4	70.6	78.3		.12
	Prior CABG^c^	47.1	46.7	47.1	47.2		.99
	Atrial fibrillation	15.9	22.2	17.2	13.2		.095
	Depression	22.6	33.3	25.6	17.3		.002
	Diabetes	34.2	37.8	34.0	33.3		.73
	Sleep apnea	29.6	28.9	31.1	28.6		.81
	Cancer	7.4	8.9	7.1	7.2		.85
	Osteoarthritis	24.3	16.7	29.8	22.3		.024
	Erectile dysfunction	50.7	56.5	50.0	49.6		.62
	PVD^d^	13.0	20.0	12.2	11.6		.10

^a^ CHD: coronary heart disease

^b^ PCI: percutaneous coronary intervention

^c^ CABG: coronary artery bypass grafting

^d^ PVD: peripheral vascular disease

**Table 3:** General care: overall and according to reported angina frequency.

Variable		Overall N=646	Daily n=90	Weekly n=238	None/monthly n=318	*P* value
Angina diagnosis, mean years ± SD		9.6 (8.8)	7.5 (7.4)	9.9 (9.2)	10.0 (8.7)	.026
Cardiology visit, % yes		80.8	73.3	84.0	80.5	.088
	If yes, discussed angina	81.8	87.9	89.0	74.6	<.001
Medication type, %	Aspirin	94.1	87.8	96.6	94.0	.010
	Any antiplatelet	100.0	100.0	100.0	100.0	n/a
	Statin	88.2	83.3	88.7	89.3	.29
	Any anti-anginal^a^	84.5	83.3	87.0	83.0	.42
	≥2 anti-anginals	35.5	44.4	38.7	30.5	.022
	Beta-blocker	74.5	72.2	73.5	75.8	.73
	Ca channel blocker	24.5	27.8	29.0	20.1	.041
	Long-acting nitrates	24.0	35.6	26.5	18.9	.003
	Ranolazine	11.5	15.6	13.5	8.8	.099

^a^ Any anti-anginal includes beta blockers, calcium channel blockers, long-acting nitrates, and ranolazine.

**Table 4:** Health-related quality of life: overall and by reported angina frequency.

Measure		Overall N=646	Daily n=90	Weekly n=238	None/Monthly n=318	*P* value
Seattle Angina Questionnaire (SAQ), mean (SD)	Angina frequency	75.4 (21.6)	41.0 (17.6)	66.9 (13.4)	91.5 (8.7)	<.0001
	Angina stability	52.4 (23.5)	33.6 (23.2)	49.6 (22.8)	59.7 (20.6)	<.0001
	Physical limitations^a^	64.7 (22.7)	52.7 (21.0)	59.5 (22.2)	72.1 (21.1)	<.0001
	Treatment satisfaction	77.5 (20.4)	60.6 (23.6)	73.0 (19.3)	85.7 (15.8)	<.0001
	Quality of life	59.0 (23.1)	33.5 (18.3)	54.0 (20.7)	69.9 (18.5)	<.0001
Global health		46.0 (23.1)	35.6 (22.8)	43.9 (22.7)	50.6 (22.2)	<.0001
EQ-5D, % no problem	Mobility	59.3	43.3	55.5	66.7	.0006
	Self-Care	91.2	81.1	89.5	95.3	<.0001
	Usual Activities	61.6	37.8	55.0	73.3	<.0001
	Pain Discomfort	29.3	6.7	12.6	48.1	<.0001
	Anxiety/Depression	53.3	27.8	49.2	63.5	<.0001
Work/Physical Activity, %	Mainly sedentary	14.1	16.7	11.8	15.1	.39
	Moderate/strenuous	33.1	28.9	26.5	39.3	0.017

^a^ 21 respondents had missing SAQ Physical Limitations scores due to more than 4 missing values out of 9.

## Discussion

### Principal Findings

This anonymous angina Internet survey provides insights into the range of symptomatic individuals in the community and raises questions about their identification and treatment. We found that approximately 15% of respondents reported daily angina, and approximately 40% reported weekly angina. In both the CHD group and non-CHD group, more frequent angina was associated with worse QOL. The cardiac origin of the symptoms cannot be proven with the methods we used; however, similarities in comorbidity, treatments, and symptoms in both the CHD and non-CHD group suggest that the burden of angina is similar in both. The majority of respondents had seen a cardiologist in the prior 6 months, yet medication and revascularization seemed lower than expected given their symptomatic status. While this methodology was successful in rapid accrual of data directly from patients, responses must be viewed in context. The Internet may be able to build a bridge for symptomatic individuals to enter the clinical setting for more timely recognition and treatment of angina.

Angina in population surveys, trials, and registries also takes place through patient report without objective confirmation, which elevates the comparability of our findings [18,19]. Most of the reported angina is likely from coronary artery disease as a majority had a prior revascularization. Angina typically persists in approximately 20% of post-MI or prior revascularization patients and exists in the same proportion among a population with stable ischemic heart disease [20]. However, up to 40% of angina patients, even those with a history of CHD, who undergo angiography have nonobstructive coronary artery disease [21]. Angina in the absence of epicardial disease may come from microvascular dysfunction, spasm, and diffuse plaques [22,23]. The possible existence of angina without ischemia or epicardial disease is important, as some providers may discount symptoms in the absence of obstructive coronary disease. For respondents in this survey with a history of angina, daily angina occurred in ~15%, and weekly angina in another 40%. Some of the subjects may have nonanginal chest pain, or somatic manifestations of depression; however, the data suggest the cause is believed to be angina from the patient perspective. Therefore, to succeed in alleviating “angina” as a source of patient suffering and impaired QOL, a broader perspective on its identifying criteria seems warranted. Requiring the presence of significant angiographic disease or abnormal stress testing may be overemphasized. Angina should be an important criterion for study inclusion in its own right.

Respondents with more angina were younger, more often female, less likely to have had prior PCI or CABG, on fewer antianginal medications, and more depressed (34.1 vs. 19.6%). Only 27% of those with daily angina reported freedom from anxiety or depression on the EQ-5D survey. Comparing to similar populations from trials and registries is challenged by the inclusion of patients with other qualifying criteria such as angiographic disease or ischemia, in addition to some degree of angina. The TERISA population enrolled patients with chronic angina in addition to diabetes [24]. The use of long-acting nitrates in the TERISA population was similar to the respondents with daily or weekly angina in this survey, with most other preventive medication use being similar. The CLARIFY registry population enrolled patients with stable ischemic heart disease, of whom 20% had angina with or without documented ischemia. In the CLARIFY population, those with angina were younger, female, and had less frequent history of revascularization than those without angina [20]. We observed that antianginal medication use increased among those with more reported angina, yet only 44% with daily symptoms were on two or more of these medications [6]. Control of blood pressure, cholesterol, and weight may also be low in this group. Of those reporting daily or weekly angina, 10-15% did not discuss it with their cardiologist, making effective treatment unlikely. Angina is associated with a significant increase in CV-related death or MI across every Canadian Cardiovascular Society Angina class [20]. This association is probably due primarily to the effect of atherosclerotic coronary artery disease; however, linkages with other prognostically important disorders such as depression might also contribute. This Internet population was identified purely through patient-reported symptoms, adding a unique comparator to symptomatic populations assembled using other inclusion criteria, and underscoring that those with chronic angina are often female and younger.

It is possible that the qualifying angina in this survey population occurred between visits to providers. The identification of a population with angina (via the Internet) raises the question of access in the event of symptom return between clinic visits. Strategies such as the brief SAQ instrument for patients with coronary disease or angina as part of routine visits, or between visits, could identify those likely to benefit from treatment intensification or revascularization [25]. Despite screening, variation in the level of angina control across health care clinics is also known to exist [26]. Although the vast majority reported having health insurance, 15% of the daily angina group also reported having no cardiologist. This suggests a lost opportunity in access to health care. Regional clinic accessibility, travel limitations, or social barriers to receiving clinic-based care may be contributors in this population.

### Limitations

To participate in an Internet survey, respondents must have computer access, which may limit generalizability [27]. As responses were voluntary, individuals with a greater burden of angina may have been more motivated to participate, leading to an overestimation of the effect of angina on QOL. Demographic or clinical data on nonrespondents could not be assessed. In addition, data on clinical diagnoses and medications were based on self-report. However, profiles and angina severity are consistent with those of contemporary populations with angina in more traditional clinical studies [28]. The possibility remains that a diagnosis of angina, as required by the screener, was misunderstood. We selected the CHD cohort for the main analysis, limited to those with documented coronary disease, to address this concern. Lastly, there was no mechanism to verify unique responses; however, each IP address could only be associated with one completed survey. This study describes responses at a single point in time, so follow-up information on treatment or outcomes was not available.

### Conclusion

This survey provides a snapshot of those with angina in an online community—at a single time point, across providers and treatment stages—and finds 15% of this group experiences daily angina. This study suggests the promise of Internet surveys for assessing patient-reported symptoms, and raises the possibility that screening tools such as this could be deployed inexpensively between health care provider encounters for better angina control and potentially improved QOL.

## Appendix

Multimedia Appendix 1 Non-CHD respondents demographics, overall and by angina frequency.

Multimedia Appendix 2 Non-CHD respondents general care, overall and by angina frequency.

Multimedia Appendix 3 Non-CHD respondents health-related quality of life assessments, overall and by angina frequency.

## Abbreviations

ACC: American College of Cardiology
CABG: prior coronary artery bypass grafting
CHD: coronary heart disease
DCRI: Duke Clinical Research Institute
EQ-5D: EuroQol-5D
HRQOL: health-related QOL
MOS: Medical Outcomes Study
PCI: percutaneous coronary intervention
PVD: peripheral vascular disease
QOL: quality of life
SAQ: Seattle Angina Questionnaire
