# Supplementary material for: Assessing Quality of Life and Medical Care in Chronic Angina: An Internet Survey
Source: Interact J Med Res. 2016 Apr 28;5(2):e12. doi: 10.2196/ijmr.4971 (PMC4865655; doi:10.2196/ijmr.4971)
Supplement: Multimedia Appendix 1 [file ijmr_v5i2e12_app1.pdf]

Multimedia Appendix 1. Non-CHD Respondents demographics, overall and by angina frequency					
	Overall N=501	*Daily N=92*	*Weekly N=223*	*None/Monthly N=186*	*PValue*
**Age, mean years (SD)**	63.0 +/- 8.6	**62.2 +/- 9.3**	**62.4 +/- 8.1**	**64.1 +/- 8.9**	**0.096**
**Male, %**	222 (44.3)	**41 (44.6)**	**97 (43.5)**	**84 (45.2)**	**0.94**
**White race, %**	461 (92.0)	**82 (89.1)**	**205 (91.9)**	**174 (93.6)**	**0.44**
**Education ≥HS, %**	482 (96.2)	**86 (93.5)**	**214 (96.0)**	**182 (97.9)**	**0.19**
**Married, %**	382 (65.5)	**67 (72.8)**	**137 (61.4)**	**124 (66.7)**	**0.14**
Insurance status					
Medicare/Medicaid, %	248 (49.5)	38 (41.3)	112 (50.2)	98 (52.7)	0.19
Private/Employer, %	296 (59.1)	58 (63.0)	123 (55.2)	115 (61.8)	0.27
No Insurance, %	48 (9.6)	8 (8.7)	29 (13.0)	11 (5.9)	0 .050
Out-of-pocket prescription costs, %					
1 (almost negligible)	190 (37.9)	39 (42.4)	73 (32.7)	78 (41.9)	0.17
2	105 (21.0)	17 (18.5)	47 (21.1)	41 (22.0)	
3	102 (20.4)	18 (19.6)	45 (20.2)	39 (21.0)	
4	57 (11.4)	8 (8.7)	30 (13.5)	19 (10.2)	
5 (can't fill all meds)	47 (9.4)	10 (10.9)	28 (12.6)	9 (4.8)	
Conditions, %					
Coronary Heart Disease	34 (6.8)	5 (5.4)	18 (8.1)	11 (5.9)	0.59
Hypertension	253 (50.5)	35 (38.0)	112 (50.2)	106 (57.0)	0.012
Prior revascularization	46 (9.2)	7 (7.6)	22 (9.9)	17 (9.2)	0.82
Prior PCI	38 (7.6)	5 (5.4)	17 (7.6)	16 (8.6)	0.64
Prior CABG	14 (2.8)	3 (3.3)	7 (3.1)	4 (2.2)	0.80
**Atrial fibrillation**	**78 (15.6)**	**12 (13.0)**	**34 (15.3)**	**32 (17.2)**	**0.66**
**Depression**	**151 (30.1)**	**32 (34.8)**	**75 (33.6)**	**44 (23.7)**	**0.051**
**Diabetes**	**100 (20.0)**	**19 (20.7)**	**44 (19.7)**	**37 (19.9)**	**0.98**
**Sleep apnea**	**128 (25.6)**	**29 (31.5)**	**56 (25.1)**	**43 (23.1)**	**0.31**
**Cancer**	**35 (7.0)**	**8 (8.7)**	**16 (7.2)**	**11 (5.9)**	**0.69**
**Osteoarthritis**	**129 (25.8)**	**27 (29.4)**	**54 (24.2)**	**48 (25.8)**	**0.64**
**Erectile dysfunction**	**99 (44.6)**	**19 (46.3)**	**41 (42.3)**	**39 (46.4)**	**0.83**
**PVD**	**22 (4.4)**	**1 (1.1)**	**13 (5.8)**	**8 (4.3)**	**0.17**
