# Supplementary material for: Assessing Quality of Life and Medical Care in Chronic Angina: An Internet Survey
Source: Interact J Med Res. 2016 Apr 28;5(2):e12. doi: 10.2196/ijmr.4971 (PMC4865655; doi:10.2196/ijmr.4971)
Supplement: Multimedia Appendix 2 [file ijmr_v5i2e12_app2.pdf]

Multimedia Appendix 2. Non-CHD respondents general care, overall and by angina frequency					
	Overall N=501	*Daily N=92*	*Weekly N=223*	*None/Monthly N=186*	*P Value*
**Angina diagnosis years, mean (SD)**	6.1 (8.3)	4.5 (8.8)	5.8 (8.0)	7.1 (8.4)	
Cardiology visit, %	228 (45.5)	35 (38.0)	106 (47.5)	87 (46.8)	0.28
If yes, discussed angina	184 (36.7)	28 (30.4)	91 (40.8)	65 (35.0)	0.18
Does not have a cardiologist	90 (18.0)	24 (26.1)	38 (17.0)	28 (15.1)	0.07
Medication type, %					
Aspirin	260 (51.9)	43 (46.7)	119 (53.4)	98 (52.7)	0.54
Any antiplatelet	267 (53.3)	45 (48.9)	120 (53.8)	102 (54.8)	0.63
Statin	232 (46.3)	36 (39.1)	93 (41.7)	103 (55.4)	0.007
Any anti-anginal[a]	244 (48.7)	37 (40.2)	110 (49.3)	97 (52.2)	0.17
≥2 anti-anginals	53 (10.6)	11 (12.0)	23 (10.3)	19 (10.2)	0.89
Beta-blocker	189 (37.7)	24 (26.1)	85 (38.1)	80 (43.0)	0.023
Ca channel blocker	73 (14.6)	13 (14.1)	32 (14.4)	28 (15.1)	0.97
Long-acting nitrates	34 (6.8)	8 (8.7)	17 (7.6)	9 (4.8)	0.39
Ranolazine	9 (1.8)	5 (5.4)	3 (1.4)	1 (0.5)	0.012

[a]Any anti-anginal includes beta blockers, calcium channel blockers, long acting nitrates, and ranolazine.
