# Supplementary material for: Assessing Quality of Life and Medical Care in Chronic Angina: An Internet Survey
Source: Interact J Med Res. 2016 Apr 28;5(2):e12. doi: 10.2196/ijmr.4971 (PMC4865655; doi:10.2196/ijmr.4971)
Supplement: Multimedia Appendix 3 [file ijmr_v5i2e12_app3.pdf]

Multimedia Appendix 3. Non-CHD respondents health-related quality of life assessments, overall and by angina frequency

	Overall N=501	*Daily N=92*	*Weekly N=223*	*None/Monthly N=186*	*P Value*
Seattle Angina Questionnaire (SAQ) scale – mean (SD)					
Angina frequency	74.6 (18.1)	51.3 (13.4)	70.4 (11.4)	91.1 (8.6)	<0.001
Angina stability	47.9 (26.0)	24.7 (21.8)	46.4 (22.6)	61.0 (23.1)	<0.001
Physical limitation[a]	67.2 (24.2)	57.4 (24.2)	65.2 (22.9)	74.9 (23.6)	<0.001
Treatment satisfaction	65.5 (24.7)	48.0 (22.9)	63.5 (23.3)	76.4 (21.4)	<0.001
Quality of life	52.4 (22.5)	32.4 (18.8)	50.6 (19.6)	64.5 (19.5)	<0.001
Global health	48.3 (23.3)	43.5 (24.0)	47.0 (23.2)	52.2 (22.6)	0.006
EQ-5D, % No problem					
Mobility	61.1	56.5	60.5	64.0	0.48
Self-Care	91.6	92.4	90.6	92.5	0.61
Usual Activities	54.3	47.8	48.9	64.0	0.004
Pain Discomfort	25.8	4.4	19.3	44.1	<0.001
Anxiety/Depression	42.7	27.2	39.9	53.8	<0.001
Work/ Physical Activity questionnaire					
Mainly sedentary, %	20.6	19.6	19.3	22.6	0.96
Moderate/strenuous exercise, %	23.0	27.2	20.2	24.2	0.20

[a]20 respondents had missing SAQ Physical Limitations scores due to more than 4 out of 9 missing values. EQ-5D, EuroQol 5-item questionnaire.
